# A Novel Targeted Long-read Sequencing Approach Boosts Transcriptomic Profiling

**DOI:** 10.1093/gpbjnl/qzae090

**Published:** 2024-12-26

**Authors:** Xiaolong Tian, Rong Fan

**Affiliations:** Department of Genetics, Yale School of Medicine, New Haven, CT 06520, USA; Department of Biomedical Engineering, Yale University, New Haven, CT 06520, USA; Department of Biomedical Engineering, Yale University, New Haven, CT 06520, USA; Yale Stem Cell Center and Yale Cancer Center, Yale School of Medicine, New Haven, CT 06520, USA; Department of Pathology, Yale School of Medicine, New Haven, CT 06520, USA; Human and Translational Immunology, Yale School of Medicine, New Haven, CT 06520, USA

In a study recently published in *Nature Communications*, Wang and colleagues developed Transcript Enrichment and Quantification Utilizing Isothermally Linear-Amplified probes in conjunction with long-read sequencing (TEQUILA-seq) [1], a novel targeted alternative isoform variation sequencing technique. This method enables the economical profiling of full-length transcriptomic landscapes with high sensitivity and fidelity, facilitating the discovery of a wide array of novel transcripts and the elucidation of RNA-associated mechanisms, like tumor suppressor gene inactivation. Such capabilities hold significant implications for a broad range of basic, translational, and clinical applications.

Mechanisms such as splicing, polyadenylation, alternative promoter usage, or RNA editing significantly diversify gene output. Hence, characterizing transcript isoforms remains a challenge when using short-read RNA sequencing. Although long-read sequencing reveals complete isoform structures, combining it with targeted sequencing can enhance the throughput for examining clinically actionable genes and address current limitations such as inadequate coverage and high cost.

Wang et al. [1] now describes TEQUILA-seq, which employs nicking-endonuclease-triggered isothermal strand displacement and amplification using only limited starting oligos. This method efficiently generates large quantities of customizable biotinylated probes specifically designed to capture target transcripts, enabling the sequencing of enriched cDNAs using Oxford Nanopore Technologies due to its easy-to-implement process. The authors demonstrate high capture efficiency and specificity, along with uniform target enrichment, across a panel of genes of various sizes. They apply TEQUILA-seq to profile 468 actionable cancer genes across 40 breast cancer cell lines, discovering cancer cell subtype-specific isoforms. Many aberrant isoforms exhibit complex splicing alterations or contain premature termination codons promoting mRNA decay, which represents a novel mechanism for inactivating tumor suppressors.

TEQUILA-seq surmounts the significant bottlenecks associated with targeted capture in long-read sequencing, including high input RNA requirements and cost-prohibitive probe synthesis which in turn facilitates the creation of custom panels for research topics of interest. It also preserves the relative proportions of transcript isoforms, crucial for accurate interpretation of the transcriptome. Importantly, the high multiplexing capacity demonstrated by introducing different barcodes points to the great potential for further integration in single-cell and spatial multi-omics sequencing [2–6] in order to map transcriptomic complexities at single-cell level and in the native tissue microenvironment. Moreover, TEQUILA-seq is expected to be compatible with not only RNA but also DNA materials and can be implemented on both long-read and short-read platforms without length-dependent biases. Taken together, these unique features of TEQUILA-seq promote a comprehensive spectrum of applications across various fields.

## Diagnostics and precision medicine

Given the critical role of alternative splicing (AS) in human health, implicated in a wide range of cancers through somatic mutations and dysregulated splicing mechanisms [7], TEQUILA-seq is uniquely suited for dissecting clinically relevant isoforms in complex cancer transcriptomes to uncover novel oncogenic transcripts. Gene fusions represent prevalent drivers in cancers, but short-read sequencing is less effective at capturing fusion junctions. TEQUILA-seq, with probe sets designed to hybridize to regions around known or suspected fusion genes, enables the multiplexed enrichment of rare fusion transcripts. This approach facilitates the identification of cancer-specific fusion isoforms, crucial for both diagnosis and therapeutic stratification. By employing synthesized probes derived from conserved regions of T-cell and B-cell receptors, the technique would further enable the selective enrichment of these repertoires from the entire transcriptome, thereby facilitating adaptive immune response profiling. TEQUILA-seq demonstrates a pivotal approach for dissecting autoimmune diseases, improving cancer treatments, and guiding the development of vaccines.

## Transcriptomic epidemiology

TEQUILA-seq can be rapidly adapted to detect emerging pathogens by using potentially broad-spectrum probes that can capture a range of pathogens or by designing new probes once partial sequences of the pathogen’s transcriptome are available. Furthermore, by sequencing entire transcriptomes of identified pathogens, TEQUILA-seq can track pathogen evolution and map transmission routes during outbreaks, which is vital for public health surveillance and response to infectious disease threats. Targeting pathogenic transcriptomes with TEQUILA-seq is well-suited to identify sequence motifs and structures of critical epitopes that elicit potent antibody responses. This would benefit the guidance of rational vaccine engineering against rapidly mutating pathogens.

## Perspectives

Looking ahead, TEQUILA-seq could be readily adapted to simultaneously sequence all non-polyadenylated transcripts through straightforward polyadenylation processes, as validated by Bai and colleagues [8]. Following this, designing targeted probes for various lengths of non-polyadenylated transcripts could significantly enhance TEQUILA-seq’s utility. Such an advancement is particularly promising for profiling long non-coding RNAs (lncRNAs), providing deep insights into their roles in gene expression and broader cellular functions. For instance, only the long isoform AS of lncRNA prostate cancer-associated transcript 19 (*PCAT19*) promotes prostate cancer growth and metastasis by interacting with the heterogeneous nuclear ribonucleoprotein A/B (HNRNPAB) complex, in contrast to its short isoform [9]. Additionally, the two isoforms of lncRNA PXN-AS1, namely PXN-AS1-L and PXN-AS1-S, exhibit antagonistic effects on paxillin (PXN) expression to modulate tumorigenesis [10].

TEQUILA-seq probes, designed to hybridize with all annotated exons of target genes, hold potential for targeted long-read DNA-seq applications directly, even though enhancements may be necessary for optimizing specific conditions and incorporating intronic targeting probes to improve the efficiency and specificity of DNA segment enrichment. While there are other established approaches for targeted DNA sequencing, such as selective sequencing based on Nanopore sequencer, which can screen target gene panels with high specificity and sensitivity without custom library preparation, these methods are necessarily require reliable reference database and complex signal mapping algorithms, or a fast base-caller to convert reference genomes into signal space [11]. Moreover, increased rejection of reads on a flow cell would critically reduce both sequencing yield and observed enrichment, rendering these approaches unsuitable for profiling small-size gene panels [11]. This feasibly extended application of TEQUILA-seq to targeted DNA sequencing, with its distinct advantages, could reveal rare and previously unidentified genetic variations, such as insertions/deletions, tandem repeats, and copy number variations linked to cancers and genetic diseases. Such insights would offer new diagnostic markers and therapeutic targets, enhancing our understanding of complex traits in human diseases. Economic traits in crops and livestock often hinge on genetic variations [12,13], and TEQUILA-seq allows for precise targeting of genes, thus potentially enables the characterization of these genetic variations. By focusing on genes related to yield, disease resistance, and environmental stress tolerance, this method can identify variant traits in crop breeds that align with agricultural goals. Similarly, in livestock, TEQUILA-seq can be used to explore the genetic foundations of traits that enhance animal health and farm productivity. This targeted approach is instrumental in breeding programs and contributes to the advancement of precision agriculture.

By applying TEQUILA-seq to full-length cDNA and gDNA of interest generated from single cells, researchers can link novel cell-to-cell transcriptomic and genetic variations to cell identity, state, and function. Integrating this technique with spatial multi-omics [14] could enhance the elucidation of correlations between novel transcriptomic, genetic variations and histopathological architecture. In the future, diverse solid tumor gene panels need to be well represented in TEQUILA-seq. Subsequently, commercially available enrichment probes can be readily accessible to facilitate the development of rapid, reliable, and cost-efficient targeted long-read sequencing. As costs continue falling, population-scale TEQUILA-seq studies promise to unravel pervasive effects of genetic variations on human diseases.

## Credit author statement

**Xiaolong Tian:** Conceptualization, Investigation, Writing – original draft, Writing – review & editing. **Rong Fan:** Conceptualization, Resources, Supervision, Writing – review & editing. Both authors have read and approved the final manuscript.

## Competing interests

RF is scientific founder and advisor of IsoPlexis, Singleron Biotechnologies and AtlasXomics. The interests of RF were reviewed and managed by Yale University Provost’s Office in accordance with the university’s conflict of interest policies. XT declares no competing interests.

## References

[1] Wang F , Xu Y, Wang R, Zhang B, Smith N, Notaro A, et al TEQUILA-seq: a versatile and low-cost method for targeted long-read RNA sequencing. Nat Commun 2023;14:4760. 37553321 10.1038/s41467-023-40083-6 PMC10409798

[2] Liu Y , Yang M, Deng Y, Su G, Enninful A, Guo CC, et al High-spatial-resolution multi-omics sequencing via deterministic barcoding in tissue. Cell 2020;183:1665–81. 33188776 10.1016/j.cell.2020.10.026 PMC7736559

[3] Deng Y , Bartosovic M, Kukanja P, Zhang D, Liu Y, Su G, et al Spatial-CUT&Tag: spatially resolved chromatin modification profiling at the cellular level. Science 2022;375:681–6. 35143307 10.1126/science.abg7216 PMC7612972

[4] Deng Y , Bartosovic M, Ma S, Zhang D, Kukanja P, Xiao Y, et al Spatial profiling of chromatin accessibility in mouse and human tissues. Nature 2022;609:375–83. 35978191 10.1038/s41586-022-05094-1 PMC9452302

[5] Liu Y , DiStasio M, Su G, Asashima H, Enninful A, Qin X, et al High-plex protein and whole transcriptome co-mapping at cellular resolution with spatial CITE-seq. Nat Biotechnol 2023;41:1405–9. 36823353 10.1038/s41587-023-01676-0 PMC10567548

[6] Zhang D , Deng Y, Kukanja P, Agirre E, Bartosovic M, Dong M, et al Spatial epigenome-transcriptome co-profiling of mammalian tissues. Nature 2023;616:113–22. 36922587 10.1038/s41586-023-05795-1 PMC10076218

[7] Zhang Y , Qian J, Gu C, Yang Y. Alternative splicing and cancer: a systematic review. Signal Transduct Target Ther 2021;6:78. 33623018 10.1038/s41392-021-00486-7 PMC7902610

[8] Bai Z , Zhang D, Gao Y, Tao B, Zhang D, Bao S, et al Spatially exploring RNA biology in archival formalin-fixed paraffin-embedded tissues. Cell 2024;187:6760–79. 39353436 10.1016/j.cell.2024.09.001 PMC11568911

[9] Hua JT , Ahmed M, Guo H, Zhang Y, Chen S, Soares F, et al Risk SNP-mediated promoter-enhancer switching drives prostate cancer through lncRNA *PCAT19*. Cell 2018;174:564–75. 30033362 10.1016/j.cell.2018.06.014

[10] Yuan JH , Liu XN, Wang TT, Pan W, Tao QF, Zhou WP, et al The MBNL3 splicing factor promotes hepatocellular carcinoma by increasing PXN expression through the alternative splicing of lncRNA-PXN-AS1. Nat Cell Biol 2017;19:820–32. 28553938 10.1038/ncb3538

[11] Payne A , Holmes N, Clarke T, Munro R, Debebe BJ, Loose M. Readfish enables targeted nanopore sequencing of gigabase-sized genomes. Nat Biotechnol 2021;39:442–50. 33257864 10.1038/s41587-020-00746-x PMC7610616

[12] Gong Y , Li Y, Liu X, Ma Y, Jiang L. A review of the pangenome: how it affects our understanding of genomic variation, selection and breeding in domestic animals? J Anim Sci Biotechnol 2023;14:73. 37143156 10.1186/s40104-023-00860-1 PMC10161434

[13] Morrell PL , Buckler ES, Ross-Ibarra J. Crop genomics: advances and applications. Nat Rev Genet 2012;13:85–96. 10.1038/nrg3097 22207165

[14] Baysoy A , Bai Z, Satija R, Fan R. The technological landscape and applications of single-cell multi-omics. Nat Rev Mol Cell Biol 2023;24:695–713. 37280296 10.1038/s41580-023-00615-w PMC10242609

